# Cholesterol Oxidase Is Indispensable in the Pathogenesis of *Mycobacterium tuberculosis*

**DOI:** 10.1371/journal.pone.0073333

**Published:** 2013-09-09

**Authors:** Magdalena Klink, Marta Brzezinska, Izabela Szulc, Anna Brzostek, Michal Kielbik, Zofia Sulowska, Jaroslaw Dziadek

**Affiliations:** Institute of Medical Biology, Polish Academy of Sciences, Lodz, Poland; University of Maryland, United States of America

## Abstract

Despite considerable research effort, the molecular mechanisms of *Mycobacterium tuberculosis* (Mtb) virulence remain unclear. Cholesterol oxidase (ChoD), an extracellular enzyme capable of converting cholesterol to its 3-keto-4-ene derivative, cholestenone, has been proposed to play a role in the virulence of Mtb. Here, we verified the hypothesis that ChoD is capable of modifying the bactericidal and pro-inflammatory activity of human macrophages. We also sought to determine the contribution of complement receptor 3 (CR3)- and Toll-like receptor 2 (TLR2)-mediated signaling pathways in the development of macrophage responses to Mtb. We found that intracellular replication of an Mtb mutant lacking a functional *choD* gene (Δ*choD*) was less efficient in macrophages than that of the wild-type strain. Blocking CR3 and TLR2 with monoclonal antibodies enhanced survival of Δ*choD* inside macrophages. We also showed that, in contrast to wild-type Mtb, the Δ*choD* strain induced nitric oxide production in macrophages, an action that depended on the TLR2, but not the CR3, signaling pathway. Both wild-type and mutant strains inhibited the production of reactive oxygen species (ROS), but the Δ*choD* strain did so to a significantly lesser extent. Blocking TLR2-mediated signaling abolished the inhibitory effect of wild-type Mtb on ROS production by macrophages. Wild-type Mtb, but not the Δ*choD* strain, decreased phorbol myristate acetate-induced phosphorylation of extracellular signal-regulated kinases 1 and 2 (ERK1/2), which are involved in both TLR2- and CR3-mediated signaling pathways. Our finding also revealed that the production of interleukin 10 by macrophages was significantly lower in Δ*choD*-infected macrophages than in wild-type Mtb-infected macrophages. However, tumor necrosis factor-α production by macrophages was the same after infection with mutant or wild-type strains. In summary, we demonstrate here that ChoD is required for Mtb interference with the TLR2-mediated signaling pathway and subsequent intracellular growth and survival of the pathogen in human macrophages.

## Introduction

The initial immune response against *Mycobacterium tuberculosis* (Mtb) starts with recognition and ingestion of mycobacteria by alveolar-resident macrophages. A number of receptors present on the cell surface of macrophages, including the mannose receptor, Toll-like receptors (TLRs) and complement receptors (CR), have been implicated in the recognition and/or uptake of mycobacteria. Phagocytosis of Mtb can involve uptake of tubercle bacilli after opsonization with serum complement as well as non-opsonic ingestion. Recognition of a specific cell wall structure of mycobacteria by TLR2 results in recruitment of adaptor protein MyD88 (myeloid differentiation primary response) to the Toll/interleukin-1 receptor (TIR) domain of TLR2, followed by recruitment of IL-1 receptor-associated kinase (IRAK)-1 and -4, which in turn leads to the phosphorylation of target signaling proteins, including MAPKs (mitogen-activated protein kinases), PI3K (phosphoinositide 3-kinase), and NF-κB (nuclear factor-κB). Signals initiated by the interaction of Mtb with TLR2 result in the induction of inflammatory and antimicrobial responses of innate immune cells [1]–[3].

Complement receptor 3 (CR3), also known as integrin α_M_β_2_, CD11b/CD18 and Mac-1, is a heterodimeric surface receptor that recognizes mycobacterial cell wall structures (non-opsonic phagocytosis) and Mtb coated with C3b or iC3b (opsonic phagocytosis). After iC3b binding, the β-subunit of CR3 mediates activation of Src-family tyrosine kinases, which subsequently phosphorylate phospholipase C. Non-opsonic binding of Mtb is important during primary infection owing to the limited presence of complement components in the alveolar space. Mtb cell wall structures are recognized by the lectin-like domain of the CR3 α-subunit. It has been reported that neither opsonic nor non-opsonic phagocytosis of Mtb via CR3 induces killing of Mtb [4], [5].

Ingestion of Mtb by macrophages induces a variety of intracellular antimicrobial mechanisms, including production of the bactericidal agents, reactive oxygen species (ROS) and reactive nitrogen intermediates (RNIs), as well as cytokines, such as tumor necrosis factor-α (TNF-α) and interleukin 10 (IL-10), that contribute to regulation of immune cell responses [6]–[8].

It is well known that Mtb is able to accumulate, and/or degrade cholesterol and use it as a source of carbon and energy and cholesterol utilization is an important determinant of Mtb survival in macrophages [9]–[14]. The initial step in cholesterol degradation is its oxidation and isomerization to cholestenone. This process is mediated by cholesterol oxidase (ChoD) and/or hydroxysteroid dehydrogenase (HsdD) [11], [15]. 3β-hydroxysteroid:oxygen oxidoreductase, commonly known as Chox, is a flavoenzyme found in a wide range of bacteria. In some bacterial species, ChoD is an extracellular enzyme that appears to be present as both secreted and cell-surface–associated forms [16]–[20]. We demonstrated previously that the Mtb H37Ra mutant strain lacking the gene encoding ChoD grows slower than wild-type H37Ra in peritoneal macrophages, lungs, and spleens of mice [21]. On the other hand, ChoD does not appear to be essential for cholesterol degradation by mycobacteria [15], [22], [23].

Here, we investigated the functional responses of human macrophages to the wild-type Mtb H37Rv strain and a mutant in which the native *choD* gene is deleted (*ΔchoD*). To this end, we assessed the interaction of the mutant with macrophages, which underlies *in vitro* recognition by phagocytes and is required for subsequent responses; examined the intracellular growth of bacteria, the production of nitric oxide (NO), ROS, and the cytokines TNF-α and IL-10 by macrophages; and determined the role of TLR2- and CR3-mediated signaling pathways in the response of macrophages to infection with wild-type and mutant strains.

## Materials and Methods

### Chemicals and Antibodies

Trypsin/EDTA (1×, 0.05% solution) and RPMI-1640 medium containing 1 mM sodium pyruvate, Dulbecco’s phosphate buffered saline (D-PBS), and Hanks’ balanced salt solution (HBSS) were purchased from Gibco (Scotland). Middlebrook OADC enrichment, Middlebrook 7H9 broth, and Middlebrook 7H10 agar were obtained from Becton Dickinson (USA). Phorbol 12-myristate 13-acetate (PMA), bovine serum albumin (BSA), fluorescein isothiocyanate (FITC), propidium iodide (PI), Triton X-100, mouse IgG2a anti-β-actin, NaF, ethylene glycol-bis(2-aminoethylether)-*N,N,N′,N′*-tetraacetic acid (EGTA), ethylenediaminetetraacetic acid (EDTA), NaCl, phenylmethylsulfonyl fluoride (PMSF), Tris, 2-mercaptoethanol (2-ME), Tween-20, Tween-80, IRAK1/4 inhibitor30% formaldehyde (FA) solution, horseradish peroxidase (HRP), Trypan Blue, and luminol were purchased from Sigma-Aldrich (USA). Protein-free Tris-buffered saline (TBS) blocking buffer, 10× Tris/glycine/SDS (sodium dodecyl sulfate) buffer, 20× Tris-buffered saline and 100× Halt protease and phosphatase inhibitor cocktail were obtained from Thermo Scientific (USA). DC Protein Assay Kit, Mini-Protean TGX Precast Gels (10%), Trans-Blot Turbo Transfer Pack PVDF (polyvinylidene fluoride), and Precision Plus Western C standard were obtained from BioRad (USA). Human type AB serum (off the clot) and fetal bovine serum (FBS) were purchased from PAA Laboratories (Austria). Mouse IgG2a anti-human TLR2 (sodium/azide free), phycoerythrin (PE)-conjugated mouse IgG2aκ isotype control, and PE-conjugated mouse IgG2a anti-TLR2 antibodies were obtained from Imgenex (USA). PE-conjugated mouse IgG1κ anti-human CR3, NA/LE mouse IgG1κ anti-human CR3 (CD11b/Mac-1; sodium/azide free), PE-conjugated NA/LE mouse IgG1κ isotype control, and NA/LE mouse IgG1κ isotype control (sodium/azide free) were purchased from BD Pharmingen (USA). Rabbit polyclonal anti-MAPK, rabbit polyclonal anti-phospho-ERK1/2 (Thr202/Tyr204), HRP-conjugated goat anti-rabbit IgG (H+L), and HRP-conjugated goat anti-mouse IgG (H+L) were obtained from Life Technologies (USA). Human IL-10 and Human TNF-α Quantikine ELISA (enzyme-linked immunosorbent assay) kits were purchased from R&D Systems (USA).

### Bacterial Growth Conditions

Wild-type, mutant, and complemented strains were grown in Middlebrook 7H9 broth supplemented with 10% OADC enrichment and 0.05% Tween-80 (in roller bottles) for 4–6 days to reach an optical density value of 1 at 600 nm (OD_600_). A portion of the bacterial culture was suspended in Middlebrook 7H9 broth (approximately 1×10^9^ cells/ml) and labeled with 100 µg/ml of FITC for 2 hours at room temperature with gentle agitation in the dark. Bacteria were then washed once with Middlebrook 7H9 supplemented with 4% BSA and twice with Middlebrook 7H9 broth without BSA [24], [25]. Thereafter, unlabeled and FITC-labeled bacteria were resuspended in Middlebrook 7H9 broth, divided into equal portions, and stored at −85°C. After 1 week, one portion of unlabeled and one of FITC-labeled bacteria were thawed, and colony-forming assays were used to determine the number of bacteria (CFUs).

Prior to infection of macrophages, bacteria were thawed, washed twice in RPMI-1640 medium, and then opsonized (or not) by incubating with 20% human AB serum in RPMI-1640 medium for 30 minutes at 37°C with gentle agitation. After opsonization, bacteria were washed once with RPMI-1640 medium. Opsonized and non-opsonized bacteria were resuspended in culture medium (CM; see below), and clumps were disrupted by multiple passages through a 25-gauge needle. Serial dilutions of bacteria were prepared in CM.

### Cell Culture

In this study, we used the differentiated human monocyte-macrophage cell line THP-1 (ACTC TIB-202; USA) as a model for macrophages. The limited number of monocyte-derived macrophages that can be obtained from blood samples is a substantial obstacle to their use in our protocols, which require very large numbers of cells. THP-1 cells were cultured in CM consisting of RPMI-1640 supplemented with 1 mM sodium pyruvate, 10% FBS, 0.05 mM 2-ME, 100 U/ml of penicillin, and 100 µg/ml of streptomycin at 37°C in a humidified 5% CO_2_ atmosphere. Monocytes were differentiated into macrophages as described previously [14] by incubating with PMA (20 ng/ml) for 24 hours (37°C/5% CO_2_). The ability of these macrophages to adhere to plastic dishes (an indicator of monocyte differentiation to macrophages) was examined under a light microscope. The macrophage-like phenotype of cells was also examined by assessing CD14 expression, as we described previously [14]. After incubation with PMA, CM was removed and macrophages were infected with bacteria. Macrophages infected with bacteria were always cultured in CM without antibiotics.

### Expression of TLR2 and CR3 on Macrophages

Macrophages were detached from plates using a trypsin-EDTA solution (2–5 minutes, 37°C/5% CO_2_); trypsin was subsequently neutralized by adding RPMI-1640 medium containing 10% FBS. Cells were then centrifuged (130× g, 6 minutes) and resuspended in D-PBS supplemented with 1% FBS. The viability of cells was determined by Trypan Blue exclusion and shown to be approximately 95%. Before staining with anti-TLR2 monoclonal antibody (mAb), crystallizable fragment receptors (FcRs) were blocked in D-PBS containing 10% human AB serum for 15 minutes at room temperature to prevent nonspecific antibody binding. Thereafter, cells were washed twice in D-PBS containing 1% FBS and stained with 10 µg/ml of PE-conjugated anti-TLR2 mAb (or 10 µg/ml appropriate IgG2a isotype control) or 20 µl PE-conjugated anti-CR3 mAb (or 20 µl appropriate IgG1 isotype control) for 30 minutes at 4°C. Cells were then washed twice, resuspended in 200 µl of D-PBS containing 1% FBS, 1% paraformaldehyde (PFA) and sodium azide, and stored at 4°C until FACS (fluorescence-activated cell sorting) analysis.

The appropriate concentration of anti-TLR2 and anti-CR3 antibodies that completely blocked the expression of TLR2 and CR3 on cells was determined by adding different mAb concentrations (10, 25, and 35 µg/ml or 25, 35, 45 and 55 µg/ml, respectively) to macrophages and incubating for 1 hour (37°C/5% CO_2_). Macrophages were then stained with PE-conjugated anti-TLR2 mAb, PE-conjugated anti-CR3 mAb or isotype controls, as described above.

All samples were examined with a FACS LSR II BD flow cytometer (Becton Dickinson, USA) equipped with BD FACSDiva Software. The results are presented as median fluorescence intensity (MFI), which correlates with the surface expression of the target molecule.

### Ingestion of Bacteria

THP-1 cells (1×10^5^ cells/well) were distributed into 8-well Permanox chamber slides (Nunc, Denmark) and differentiated into macrophages. Cells were then pre-treated with 10 µM IRAK1/4 inhibitor or 35 µg/ml of anti-TLR2 or 55 µg/ml anti-CR3 blocking mAb for 1 hour (37°C/5% CO_2_) or left untreated (as indicated in figures). Thereafter, macrophages were infected with opsonized or non-opsonized, FITC-labeled, wild-type, mutant (Δ*choD*), or complemented (Δ*choD*-*choD*) strains at a multiplicity of infection (MOI) of 10 for 2 hours (37°C/5% CO_2_). Non-ingested bacteria were removed by extensively washing macrophages with warm HBSS. Fluorescence quenching by extracellular bacteria was removed by adding an equal volume of 2 mg/ml Trypan Blue solution. Phagocytes were fixed by incubating with 3% PFA for 15 minutes (37°C/5% CO_2_) and washed twice with HBSS. The number of infected macrophages and the number of bacteria engulfed per macrophage were determined by fluorescence microscopic examination (Nikon ECLIPSE TE 2000 U). In all cases, 200 macrophages were counted.

### Intracellular Growth of Bacteria

THP-1 cells (1×10^5^ cells/well) were distributed into 24-well plates (Nunc), differentiated into macrophages, and then pretreated with 10 µM IRAK1/4 inhibitor, 35 µg/ml of anti-TLR2 mAb or 55 µg/ml of anti-CR3 blocking mAb, as described above. Macrophages were then infected with opsonized or non-opsonized wild-type, Δ*choD,* or Δ*choD*-*choD* Mtb strains at an MOI of 1. Non-ingested bacteria were removed by extensively washing with warm HBSS. Fresh CM and IRAK1/4 inhibitor or antibodies (as indicated) were added, and cells were cultured for 6 days. On day 0 and days 2, 4 and 6 post-infection, macrophages were lysed with 1 ml of 0.1% Triton X-100. Appropriate dilutions of cell lysates were plated onto Middlebrook 7H10 agar supplemented with 10% OADC. After 21 days of culture, CFUs were counted. The data are presented as fold increase in CFUs/ml, calculated as CFUs/ml on day 6 divided by CFUs/ml on day 0.

### NO and ROS Production

THP-1 cells (1×10^5^ cells/well) were distributed into 96-well plates (Nunc) and differentiated into macrophages. After that, macrophages were pre-treated with 10 µM IRAK1/4 inhibitor or 55 µg/ml of anti-CR3 mAb, or were left untreated (see above), as indicated in figures. Cells were then infected for 2 hours with opsonized or non-opsonized wild-type, Δ*choD,* or Δ*choD*-*choD* strains at an MOI of 10. Extracellular bacteria were removed by extensively washing macrophages with warm HBSS. Macrophages with ingested bacteria were cultured for 24 hours (ROS production) or 48 hours (NO production).

In the case of ROS production, after 24 hours of culturing, the supernatants were harvested and 1 µg/ml of PMA, 40 U of HRP (to initiate ROS production), 1 mM luminol (to enhance chemiluminescence (CL), and HBSS were added to cells. CL was recorded over 4 hours at 5-minute intervals. Data were acquired as relative light units (RLU), and the area under the curve of CL versus assay time (total RLU) was calculated. Data are presented as the percentage inhibition of ROS production, calculated according to the formula: 1– (total RLU for cells infected with bacteria and stimulated with PMA/total RLU for cells stimulated with PMA) ×100.

The presence of nitrite (stable metabolite of NO) in the culture supernatants of macrophages infected with bacteria was detected using the Griess reagent. Nitrite concentration was calculated from a standard curve using sodium nitrite as a reference. OD was determined using a Multiscan RC ELISA reader (Labsystem, Finland).

### Western Blot Analysis of ERK1/2

THP-1 cells (5×10^6^ cells/well) were distributed into 24-well plates (Nunc), differentiated into macrophages, and then infected with bacteria (MOI = 10) as described above. Thereafter, in one set of experiments, macrophages were incubated with 1 µg/ml of PMA for 2 hours or left untreated. In a second set of experiments, macrophages were cultured for 24 hours and then stimulated with PMA. After treatment with PMA, cells were detached, centrifuged (1 minute, 12,000× g), and lysed in lysis buffer (1% Triton-X 100, 20 mM Tris, 150 mM NaCl, 1 mM EDTA, 1 mM EGTA, 1 mM PMSF) containing 1× Halt protease and phosphatase inhibitor cocktail by incubating for 30 minutes on ice. The protein concentration in each lysate was determined using a DC Protein Assay Kit.

Cell lysates containing equal amounts of protein were run on 10% Mini-Protean TGX Precast Gels along with a molecular weight standard. The proteins were transferred to PVDF membranes using Trans Blot Turbo (Bio-Rad, USA) at 2.5 A (∼25 V) for 10 minutes. The membranes were blocked with protein-free (TBS) blocking buffer for 20 minutes and then incubated with primary anti-MAPK Abs (1∶1000), anti-phospho-ERK1/2 Abs (1∶1000), or anti-β-actin Abs (1∶4000) at room temperature for 1 hour. After washing five times in 2× TBS/Tween-20, membranes were incubated with HRP-conjugated goat anti-rabbit IgG (H+L) (1∶4000) or HRP-conjugated goat anti-mouse IgG (H+L) (1∶4000) at room temperature for 1 hour, and then washed. Immunoreactive proteins were visualized using an enhanced chemiluminescence system (Thermo Scientific, USA). Densitometric analyses of blots and analyses of visualized bands were performed using a FluoroChem MultiImage FC Cabinet (Alpha Innotech Corporation, San Leandro, CA, USA) and Alpha Ease FC software 3.1.2. The results are presented as the optical density intensity (ODI) of the area under each band’s peak.

### TNF-α and IL-10 Production

THP-1 cells (1×10^6^ cells/well) were distributed into 24-well plates (Nunc), differentiated into macrophages, and then infected with bacteria (MOI = 10), as described above, and cultured for 24 hours. The presence of IL-10 and TNF-α in the culture supernatants was assessed using Quantikine ELISA kits. The sensitivities of IL-10 and TNF-α assays were 3.9 and 1.6 pg/ml, respectively.

### Statistical Analysis

Data are presented as means ± SEMs. Statistical significance was verified using nonparametric Wilcoxon’s signed-rank or Mann-Whitney *U* tests. The Statistica 8.0 (StatSoft, Poland) software package was used for statistical calculations. Statistical significance was defined as p ≤0.05.

## Results

### The Surface Expression of TLR2 and CR3 on Macrophages

**Figure 1:**
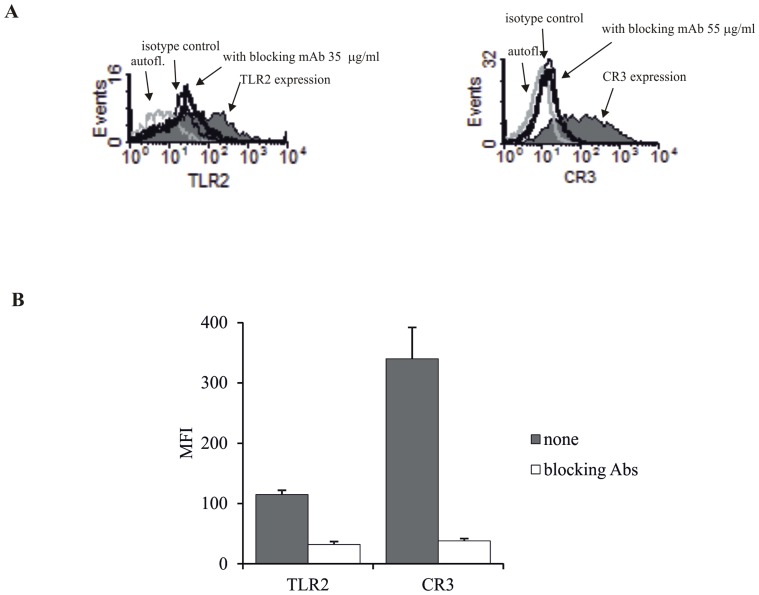
Flow cytometry analysis of TLR2 and CR3 expression on macrophages. Macrophages were incubated with 35 µg/ml of anti-TLR2 blocking mAb or 55 µg/ml of anti-CR3 blocking mAb, or were left untreated, for 1 hour. Cells were then stained with PE-conjugated anti-TLR2 mAb, PE-conjugated anti-TLR2 mAb, or specific isotype controls for 30 minutes. (A) Representative histograms show TLR2 and CR3 expression on macrophages treated or untreated with blocking antibodies. (B) The graph presents mean values of MFI ± SEM of receptors on macrophages, untreated or treated with blocking antibodies. Data are presented from eight independent experiments.

The expression levels of TLR2 and CR3, determined by flow cytometry as MFI values, were 115±7 and 340±43, respectively. We also determined the concentration of blocking anti-TLR2 and anti-CD3 mAbs sufficient to neutralize surface expression of each receptor. As shown in Figure 1, we found that after pre-incubation of macrophages with 35 or 55 µg/ml of blocking mAbs, surface expression of TLR2 (MFI = 32±5) and CR3 (MFI = 38±4) was virtually undetectable.

### Phagocytosis of Mycobacteria

**Figure 2:**
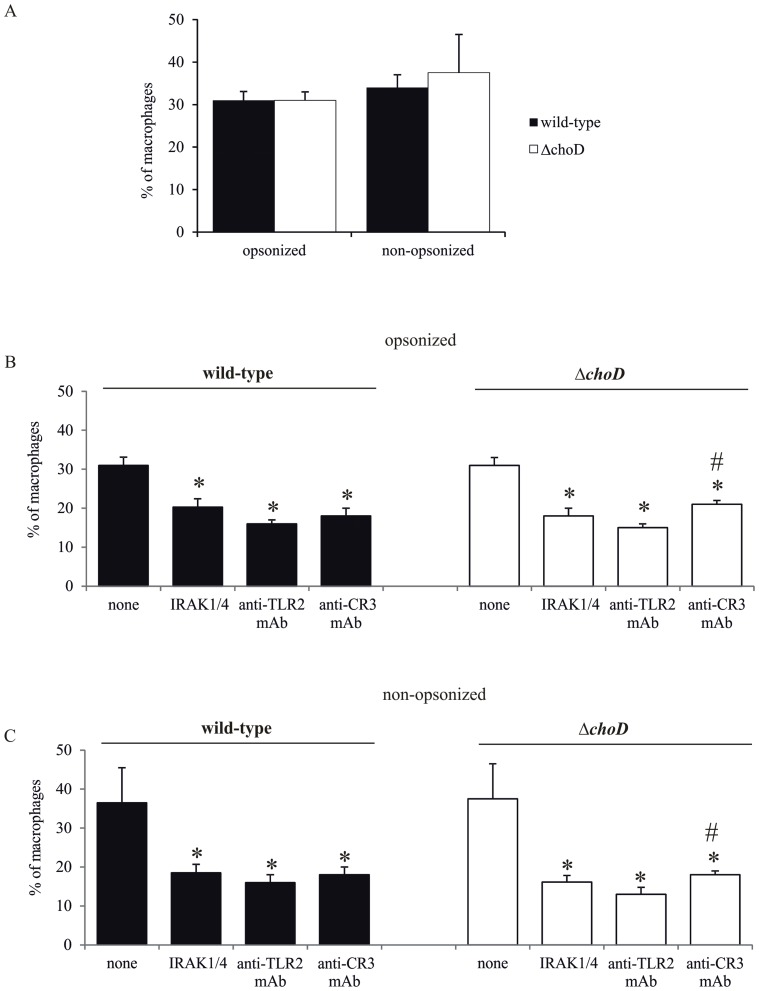
Percentage of macrophages that ingest Mtb. (A) Macrophages were infected with FITC-labeled wild-type *or* Δ*choD* strains for 2 hours. (B and C) Macrophages were treated with IRAK1/4 inhibitor, anti-TLR2 mAb, or anti-CR3 mAb for 1 hour, and then were infected with (B) opsonized or (C) non-opsonized FITC-labeled wild-type or Δ*choD* strains for 2 hours. In all samples, macrophages with ingested bacteria were counted using a fluorescence microscope. All data are presented as the percentage of macrophages involved in phagocytosis, expressed as means ± SEMs (*p≤0.02, Mtb strain vs. Mtb strain+IRAK1/4 inhibitor or anti-TLR2 mAb or anti-CR3 mAb, # p≤0.03, Δ*choD*+anti-CR3 mAb vs. Δ*choD*+anti-TLR2 mAb,; Mann-Whitney *U* test). Data are presented from five independent experiments. Each experiment was carried out in duplicate.

Three strains of Mtb H37Rv were used in each experiment: wild-type Mtb; a site-directed mutant in which the native *choD* gene was replaced with a truncated, non-functional *choD* (Δ*choD*); and a complemented strain carrying an intact *choD* gene under control of a P_hsp_ promoter introduced into the *attB* site (Δ*choD-choD*), engineered as described previously (25). We found that the percentage of macrophages ingesting wild-type and Δ*choD* Mtb strains ranged from 25% to 40%, although there was no significant difference in the percentage of macrophages that ingested either strain (Fig. 2A). We also noted that inhibition of TLR2- or CR3-mediated signaling pathways decreased the efficiency of phagocytosis that manifested as a decrease in the percentage of macrophages that took up opsonized or non-opsonized both wild type and Δ*choD* Mtb strains (Fig. 2B and C). Notably, the anti-TLR2 mAb decreased phagocytosis to a greater degree than the anti-CR3 mAb.

### Intracellular Growth of Wild-type, *ΔchoD*, and *ΔchoD-choD* Strains

**Figure 3:**
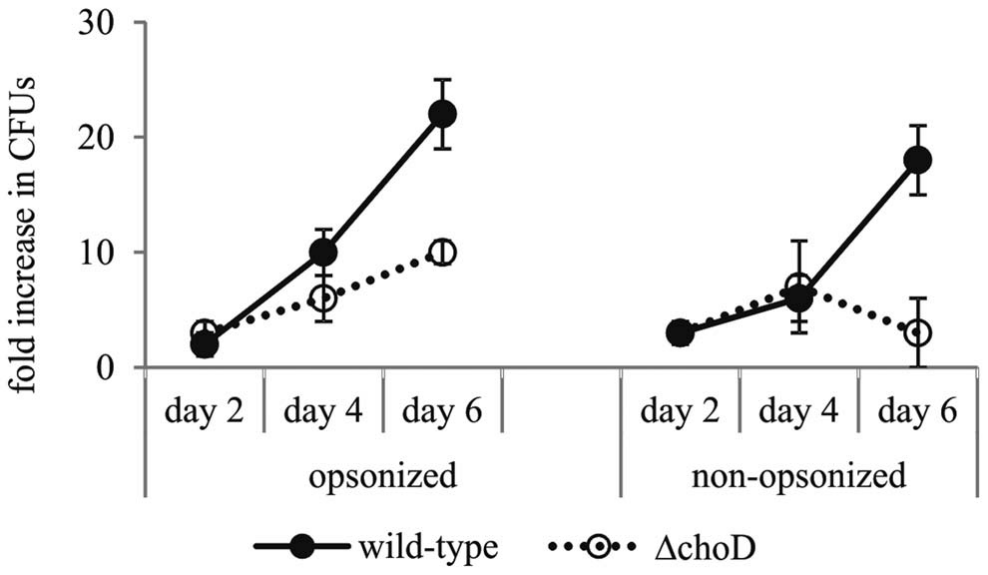
Time-dependent survival of Mtb in macrophages. Macrophages were infected with wild-type or Δ*choD* strains for 2 hours and washed with HBSS. On the day of infection and after 2, 4 or 6 days in culture, macrophages were lysed with Triton X-100 and cell lysates were plated onto agar plates. After 21 days of culture, CFUs were counted. The data are presented as fold increase in CFUs, expressed as means ± SEMs (n = 3). Data are from five independent experiments.

A survey of bacteria surviving inside macrophages, determined by colony-forming assays and expressed as fold increase in CFUs, was used to test bacterial intracellular growth. In a preliminary study, we tested the survival of wild-type and Δ*choD* strains in macrophages 2, 4 and 6 days after infection, and found that the intracellular growth of Mtb increased significantly with increasing culture duration. We also observed that the growth of wild-type and Δ*choD* strains was similar up to 4 days post-infection, but differed significantly on day 6 post-infection (Fig. 3).

**Figure 4:**
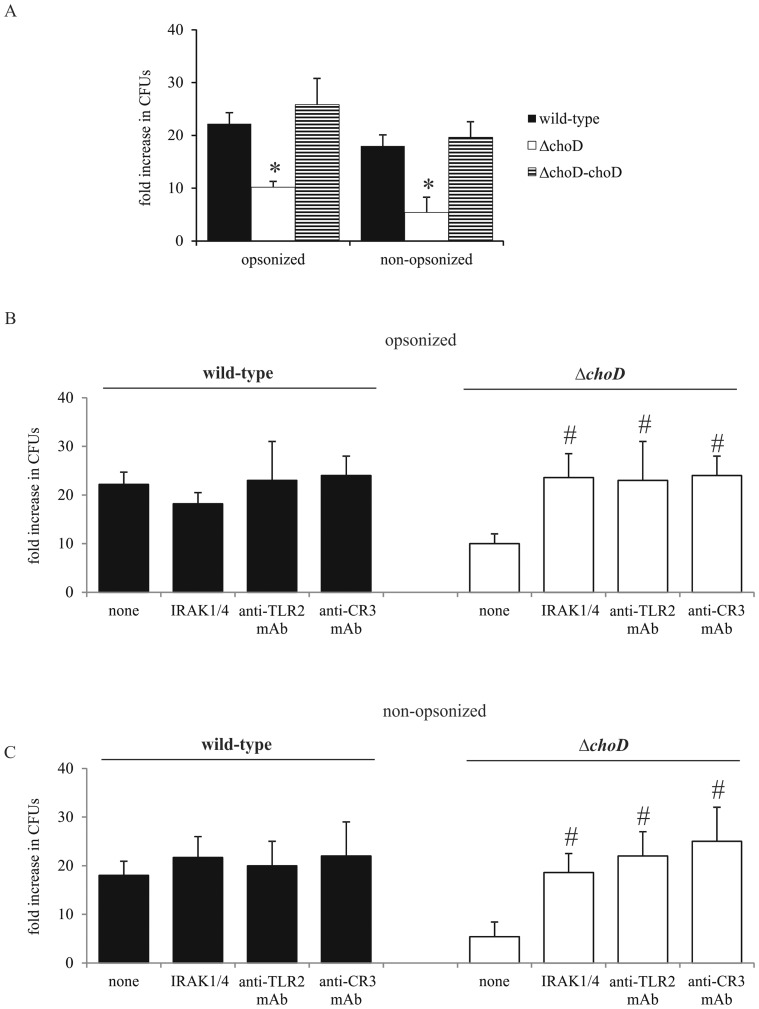
Survival of Mtb in macrophages. (A) Macrophages were infected with wild-type, Δ*choD* or Δ*choD*-*choD* strains for 2 hours without inhibitor or mAb and then washed with HBSS. (B and C) Macrophages were incubated with IRAK1/4 inhibitor, anti-TLR2 blocking mAb, or anti-CR3 blocking mAb for 1 hour prior to infection with opsonized (B) or non-opsonized (C) wild-type or Δ*choD* strains, and then washed. On the day of infection and after 6 days in culture, macrophages were lysed with Triton X-100 and cell lysates were plated onto agar plates. After 21 days of culture, CFUs were counted. The data are presented as fold increase in CFUs, expressed as means ± SEMs (*p≤0.03, Δ*choD* vs. wild-type or Δ*choD*-*choD*; #p≤0.04, Δ*choD* vs. Δ*choD*+IRAK1/4 inhibitor or Δ*choD*+anti-TLR2 mAb or Δ*choD*+anti-CR3 mAb; Mann-Whitney *U* test). Data are presented from five independent experiments. Each experiment was carried out in duplicate.

A comparison of the intracellular growth of mutant and wild-type Mtb in macrophages performed 6 days after infection showed that intracellular replication of opsonized and non-opsonized Δ*choD* strain in macrophages was significantly impaired compared to that of wild-type and complemented (Δ*choD*-*choD*) strains (Fig. 4A). Neither inhibition of the TLR2-mediated signaling pathway with blocking mAb or IRAK1/4 inhibitor nor inhibition of CR3-mediated signaling pathway with blocking mAb had a significant effect on the survival of the wild-type strain in macrophages (Fig. 4B and C). However, treatment with blocking antibodies or IRAK1/4 inhibitor significantly increased the growth of both opsonized and non-opsonized Δ*choD* in macrophages compared to macrophages not treated with mAbs (Fig. 4 B and C), indicating that the TLR2- and CR3-mediated signaling pathways are involved in limiting Δ*choD* replication in macrophages. Dimethyl sulfoxide (DMSO), used as a vehicle to prepare IRAK1/4 inhibitor solutions, at a final concentration of 0.5% had no effect on the growth of Mtb strains in macrophages. Similarly, IRAK1/4 inhibitor at the concentration of 10 µM had no significant effect on the viability of macrophages up to six days (% of viable macrophages with and without IRAK1/4 inhibitor at 6^th^ day amounted 90% and 89%, respectively).

### NO and ROS Production by Macrophages Infected with Wild-type, *ΔchoD*, or *ΔchoD-choD* Strains

**Figure 5:**
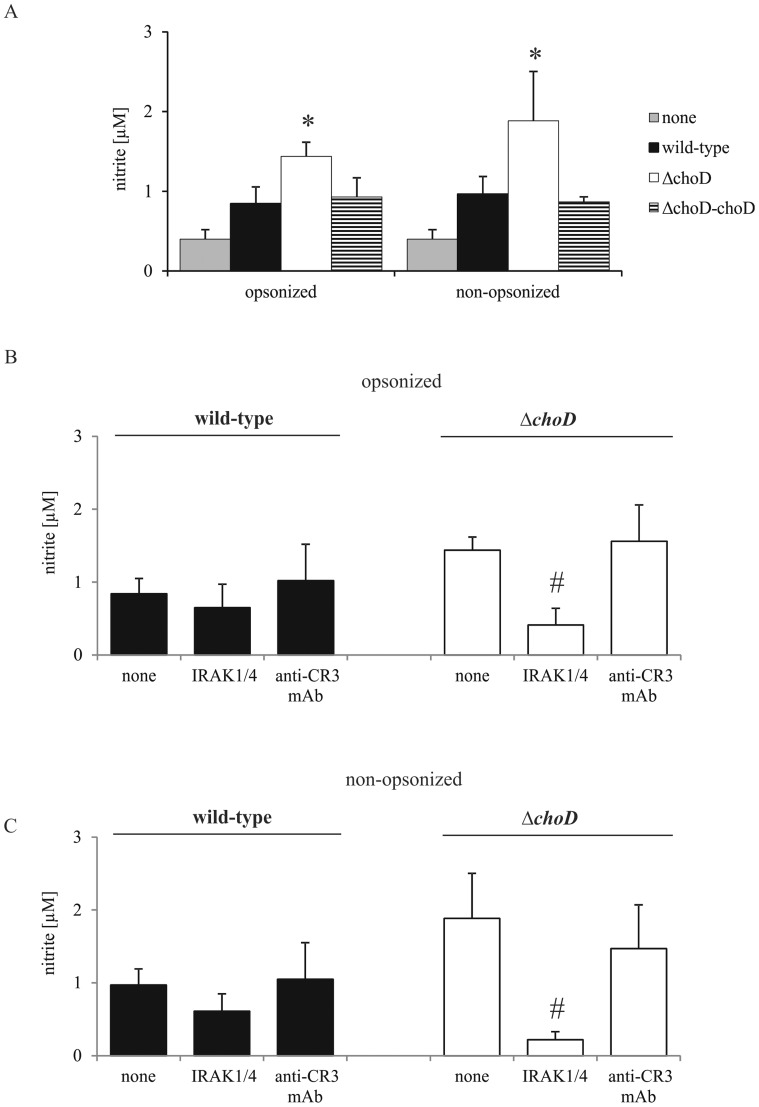
NO production by infected macrophages. (A) Macrophages were infected with wild-type, Δ*choD*, or Δ*choD*-*choD* strains for 2 hours without inhibitor or mAb and then washed with HBSS. (B and C) Macrophages were incubated with IRAK1/4 inhibitor or anti-CR3 blocking mAb for 1 hour prior to infection with opsonized (B) or non-opsonized (C) wild-type or Δ*choD* strains. After culturing for 48 hours, the concentration of nitrite, a stable metabolite of NO, was assessed in culture supernatants using the Griess reagent. The data are presented as nitrite concentration (µM), expressed as means ± SEMs (*p≤0.03, Mtb strain vs. none (macrophages in CM); Wilcoxon’s signed-rank test; #p≤0.04, Δ*choD* vs. Δ*choD*+IRAK1/4 inhibitor; Mann-Whitney *U* test). Neither IRAK1/4 inhibitor nor anti-CR3 mAb significantly influenced NO production by uninfected macrophages. Data are presented from six independent experiments. Each experiment was carried out in triplicate.

Bacteria-induced NO production by macrophages was determined 48 hours after infection, a time chosen because preliminary experiments showed that the level of nitrite (a stable metabolite of NO) was almost undetectable in 24-hour culture supernatants. We found that both opsonized and non-opsonized Δ*choD*, but not wild-type or complemented strains, induced NO production (Fig. 5A). However, *ΔchoD* failed to stimulate NO production when the TLR2-mediated signaling pathway was disrupted by IRAK1/4 inhibitor (Fig. 5B and C). In contrast, treatment of macrophages with IRAK1/4 inhibitor or anti-CR3 mAb had no effect on NO production after infection with the wild-type strain (Fig. 5 B and C). In the preliminary experiments anti-TLR2 mAb were used and the results obtained were similar as in case IRAK1/4 inhibitor (mutant strain opsonized 1.96 µM, and non-opsonized 1.85 µM; in the presence of IRAK1/4 inhibitor 0.45 µM and 0.40 µM; and in the presence of anti-TLR2 mAb 0.35 µM and 0.49 µM, respectively). In control experiments, DMSO (0.5%) alone did not affect production of NO by macrophages (0.40±0.2 vs. 0.37±0.2 µM nitrite in the presence and absence of DMSO, respectively).

**Figure 6:**
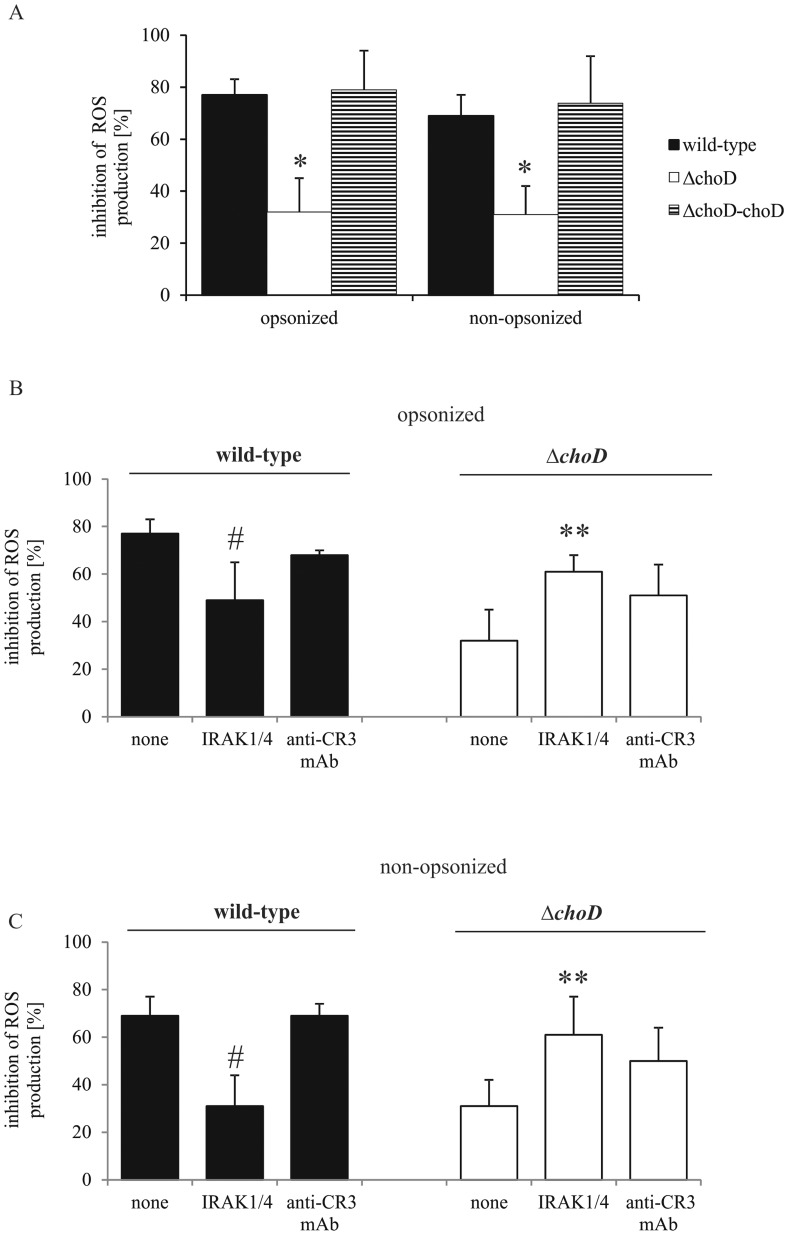
ROS production by infected macrophages. (A) Macrophages were infected with wild-type, Δ*choD*, or Δ*choD*-*choD* strains for 2 hours, washed with HBSS, and then cultured for 24 hours. (B and C) Macrophages were incubated with IRAK1/4 inhibitor or anti-CR3 mAb for 1 hour, and then were infected with opsonized (B) or non-opsonized (C) wild-type or Δ*choD* strains for 2 hours, washed with HBSS, and cultured for 24 hours. Cells were then stimulated with PMA, and ROS production was assessed using the CL assay. Data are presented as the percentage inhibition of ROS production, expressed as means ± SEMs (*p≤0.04, Δ*choD* vs. wild-type or Δ*choD*-*choD*; #p≤0.05, wild-type vs. wide-type+IRAK1/4 inhibitor; **p≤0.05, Δ*choD* vs. Δ*choD*+IRAK1/4 inhibitor; Mann-Whitney *U* test). Data are presented from five independent experiments. Every experiment was carried out in triplicate.

We found that neither the mutant strain nor wild-type Mtb influenced ROS production by non-stimulated macrophages 24 hours post-infection. However, the wild-type strain strongly inhibited the ability of macrophages to produce ROS in response to stimulation with PMA. We further found that opsonized and non-opsonized *ΔchoD* exhibited a significantly weakened ability to suppress ROS production by macrophages compared to wild-type and complemented strains (Fig. 6A). In subsequent experiments, we treated macrophages with IRAK1/4 inhibitor or anti-CR3 mAb before infection with Mtb. As shown in Figure 6B and C, IRAK1/4 inhibitor significantly reduced the ability of the wild-type strain to impair ROS production by macrophages. In contrast, the decrease in ROS production by macrophages infected with Δ*choD* was significantly greater in the presence of IRAK1/4 inhibitor than in the absence of inhibitor (Fig. 6 B and C). It was found that in the presence of IRAK1/4 inhibitor, wild-type strain had similar effect on the ROS production as mutant strain without the inhibitor. For opsonized bacteria, the percentage of ROS inhibition observed in wild-type Mtb-infected macrophages treated with IRAK1/4 inhibitor or Δ*choD*-infected macrophages without inhibitor was 49% and 31%, respectively. In the case of infection with the non- opsonized bacteria, both wild-type Mtb in the presence of the inhibitor and mutant strain without inhibitorinhibited ROS production by 31%. Treatment of macrophages with anti-CR3 mAb did not affect ROS inhibition by macrophages infected with the wild-type strain. A trend toward increased inhibition of ROS production that did not reach significance was observed in macrophages infected with the mutant strain in the presence of anti-CR3 mAb. Neither the vehicle for PMA (0.1% ethanol in HBSS) nor 0.5% DMSO in HBSS affected ROS production by macrophages. IRAK1/4 inhibitor at the concentration of 10 µM had no significant effect on the viability of macrophages after 24 hours of culture (% of viable macrophages in the presence and absence of IRAK1/4 inhibitor amounted 91% and 95%, respectively).

### Effect of Wild-type and *ΔchoD* Strains on PMA-stimulated ERK1/2 Phosphorylation

**Figure 7:**
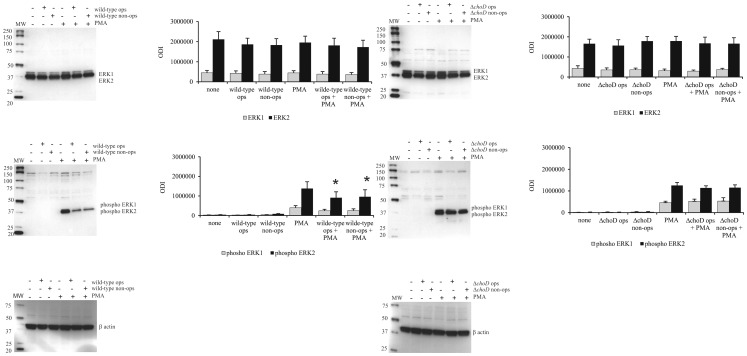
Western blot analysis of ERK1/2 expression and phosphorylation in infected macrophages. Macrophages were infected with (A) wild-type or (B) Δ*choD* strains for 2 hours, washed with HBSS, and cultured for 24 hours. Cells were then treated with PMA for 2 hours. Representative immunoblots of ERK1/2 protein level and phosphorylation status are presented. Bands were quantified by densitometric analysis. Data are presented as optical density intensity of the area under each phosphoERK1/2 band’s peak (ODI) ± SEM (*p≤0.04, PMA vs. wild-type+PMA; Mann-Whitney *U* test). Data are presented from five independent experiments.

We found that during a 2-hour phagocytosis experiment, neither wild-type nor mutant strains affected PMA-induced phosphorylation of ERK1/2 in macrophages (data not shown). Because it is known that Mtb displays a very long infection cycle time in macrophages, we tested the impact of Mtb strains on ERK1/2 phosphorylation 24 hours post-infection. These experiments showed that PMA-induced phosphorylation of ERK1/2 was significantly inhibited by opsonized and non-opsonized wild-type strain (Fig. 7A), but not by the *ΔchoD* mutant strain (Fig. 7B).

### TNF-α and IL-10 Production Response in Macrophages Infected with Wild-type, *ΔchoD*, or *ΔchoD-choD* Strains

**Figure 8:**
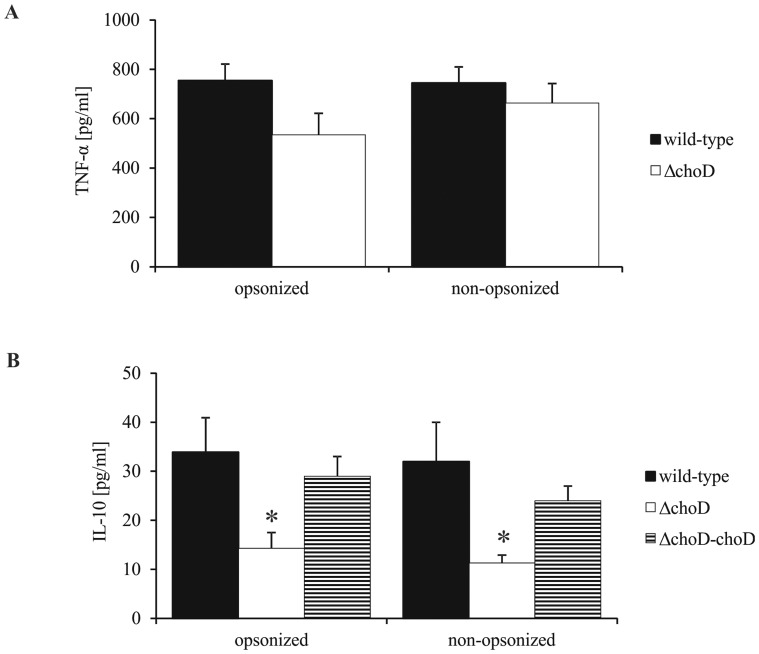
TNF-α and IL-10 production by infected macrophages. Macrophages were infected with wild-type, Δ*choD*, or Δ*choD*-*choD* strains for 2 hours, then washed with HBSS and cultured for 24 hours. The amount of released TNF-α (A) or IL-10 (B) was assessed in culture supernatants using an ELISA kit. Data are expressed as means (pg/ml) ± SEMs (*p≤0.02, Δ*choD* vs. *choD* or Δ*choD*-*choD*; Mann-Whitney *U* test). Data are presented from five independent experiments. Each experiment was carried out in duplicate.

The production of TNF-α by macrophages infected with wild-type or *ΔchoD* strains was very similar (Fig. 8A). However, Δ*choD* strain (opsonized and non-opsonized) stimulated significantly lower macrophage production of IL-10 than the wild-type strain and complemented mutant (Δ*cho*-*choD)* did (Fig. 8B). In the absence of Mtb infection, macrophages released relatively low amounts of TNF-α (10.9±0.4 pg/ml) and IL-10 (1.3±0.4 pg/ml).

## Discussion

Despite considerable research effort, the molecular mechanisms of Mtb virulence remain unclear. Pathogenic mycobacteria can affect the function of immune cells through secreted extracellular proteins or cell wall components [26]. We have previously shown that the Mtb *ΔchoD* mutant is attenuated in mice and peritoneal macrophages [21]. On the other hand, ChoD does not appear to be essential for cholesterol degradation, suggesting that the observed attenuation is not attributable to nutrient limitations during intracellular growth [15]. In the current study, we found that ChoD is required for modification of the antibacterial activity of human macrophages by Mtb. We also observed that inactivation of TLR2- or CR3-mediated signaling (with blocking mAbs) improved the growth of the *ΔchoD* mutant in macrophages to the level of the wild-type strain, suggesting that ChoD is required to engage the TLR2- and CR3-mediated signaling that results in prolonged survival of bacilli inside macrophages.

There was little difference in the engulfment of wild-type and *ΔchoD* strains by THP-1 cells, and blocking TLR2- or CR3-mediated signaling pathways decreased the ingestion of both investigated strains. However, as previously observed in a mouse model [21], the *ΔchoD* mutant appeared to be attenuated in THP-1-derived macrophages compared to wild-type Mtb. This observation was verified using the complemented mutant, Δ*choD-choD*, carrying an intact copy of *choD* introduced into the *attB* site of chromosomal DNA. Cholesterol transport/degradation mutants have previously been described as attenuated *in vivo*, suggesting an essential nutrient role of cholesterol in the pathogenic process. For example, Δ*mce4c*, an Mtb mutant defective in cholesterol transport, appears to be less virulent then the wild-type strain in a mouse model [12]. Similarly, a 3-ketosteroid 9α-hydrolase mutant is attenuated in mouse bone marrow macrophages [10],, and the side-chain–degradation mutant, Δ*fadA5*, is impaired in the late stage of mouse infection [27]. Additionally, the Δ*kstD* strain, which is unable to degrade the cholesterol ring structure, is attenuated in human macrophages differentiated from THP-1 cells [14]. It was also previously shown that the Δ*igr* strain, which is defective in degradation of the 26-propionate side chain fragment, is attenuated in mice [28], [29]. The *in vivo* and *in vitro* attenuation of these mutants was related to their inability to use cholesterol as a nutrient in the pathogenicity process and/or to their accumulation of toxic degradation intermediates [10]. On the other hand, ChoD probably does not play an essential role in cholesterol degradation [15], [22], [23]. Thus, we hypothesized that the attenuated growth of the Δ*choD* strain was not due to the inhibition of cholesterol degradation but rather was attributable to the enhanced functional response of macrophages.

Upon activation by engulfment of Mtb, phagocytes produce ROS and NO, which participate in bacteria killing and/or growth inhibition [8], [30]–[32]. The NO response of macrophages was effectively suppressed by wild-type and complemented Mtb, but not by Δ*choD*; in macrophages infected with Δ*choD,* NO overproduction remained intact. This induction of NO production by Δ*choD* was blocked by IRAK1/4 inhibitor and anti-TLR2 mAbs, but not by a mAb to CR3, demonstrating its dependence on the TLR2, but not the CR3, pathway. This finding is consistent with a previous report that demonstrated an essential role for TLR2 in inducible nitric oxide synthase (iNOS) expression in Mtb-infected macrophages [33]. Other investigators [34], [35] have reported that Mtb induces ROS production in macrophages 30 minutes post-infection. Here, we observed that PMA-stimulated ROS production was attenuated by 80% in macrophages infected with wild-type Mtb, but only by 20% in Δ*choD*-infected macrophages. Moreover, the inhibition of ROS production observed in the wild-type strain was partially blocked by IRAK1/4 inhibitor, but not by an anti-CR3 mAb. IRAK1 and −4 have been reported to interact with protein kinase C, which phosphorylates NADPH oxidase [36], [37] and can also directly phosphorylate NADPH oxidase to effectively promote the production of ROS [35]. Our data are in accord with a report that the CR3-mediated signaling pathway is not involved in NO or ROS production in Mtb-infected macrophages [38], and collectively suggest that functional ChoD in the wild-type strain acts through the TLR2-mediated signaling pathway to play an essential role in suppressing the bactericidal activity of macrophages.

Consistent test results obtained by us with the use of antibodies anti-TLR2 and IRAK1/4 inhibitor argue in favor for signaling pathways involvement through TLR2. It is known that IRAK1/4 are important mediators in signal transduction of the TLR family, including also TLR1/2/6/5/7/8/9 and they may act to potentiate the downstream signaling [3]. Therefore we cannot completely exclude the participation of other TLRs in the response of macrophages to the Mtb infection.

Activation of TLR2 results in phosphorylation of the MAPK family member ERK1/2 [39]. ERK1/2, in turn, participates in induction of iNOS expression and activity in phagocytes as well as phosphorylation of NADPH oxidase components p47^phox^ and p67^phox^, which are responsible for NO and ROS production [40]–[42]. We found here that wild-type Mtb, but not Δ*choD,* blocked the ability of PMA to induce ERK1/2 phosphorylation in macrophages, confirming that ChoD is required for Mtb to disrupt the TLR2-activated signaling pathway that leads to inhibition of the antibacterial response of macrophages. We also found that ChoD of *Nocardia erythropolis* significantly decreased PMA-stimulated phosphorylation of ERK1/2 in macrophages (data not shown).

As reported previously [43], the TLR2 pathway can be utilized by Mtb as a survival mechanism (e.g., through inhibition of phagosome maturation); however, the Mtb factor responsible for disrupting TLR2-mediated signaling has remained unknown. Our results suggest the importance of ChoD in this process. In this context, it should be noted that, during the phagocytosis process, TLR2 proteins are recruited to the macrophage phagosome [44], where they may interact with phagocytosed Mtb. Moreover, it was recently reported that Mtb is able to escape the phagosome and gain access to the cytosol of infected host macrophages [45], [46]; thus, ChoD could exert a suppressive effect on signaling proteins in the cytosol. Whether ChoD affects TLR2 signaling proteins directly or inactivates this signaling pathway through interactions with lipid rafts, as has been suggested [47], remains to be clarified.

Another possible impact of Mtb is through direct actions of the bacteria on TLR2 activity [48]. In this study, Madan-Lala and coworkers demonstrated that the cell envelope-associated serine hydrolase, Hip1, is an important Mtb virulence factor that modulates pro-inflammatory responses in Mtb-infected macrophages. The authors concluded that Hip1 limits the magnitude of macrophage responses by suppressing activation of the TLR2 signaling pathway. It has also been demonstrated that direct treatment of cells with ChoD from *Pseudomonas fluorescens* depletes cholesterol from cell membranes, thereby affecting the conformation and function of chemokine receptors in the plasma membrane [47]. It is accepted that, after penetration into macrophages, tubercle bacilli reside predominantly in a cholesterol-rich region of the cellular plasma membrane. Changes in the cholesterol level in the plasma membrane modulate the activity of proteins and receptors located in lipid rafts [49], which can determine the activity of cytosolic signaling protein, such as NF-κB [50].

The phagocytosis of Mtb initiates the production of various pro-inflammatory cytokines, including TNF-α and IL-10. IL-10 has been shown to inhibit phagosome maturation and ROS and RNI production in phagocytes [7], [51], [52]. In the current study, we observed an elevation in the level of IL-10 produced by macrophages infected with the wild-type strain compared to that in macrophages infected with the *ΔchoD* mutant. The multiplication of both strains was the same during the period of the experiment. This increase in IL-10 level in macrophages in response to infection by the wild-type strain was associated with an absence of ROS and NO production. TNF-α acts in synergy with interferon γ (IFN-γ) in the killing of Mtb through the induction of NO and ROS production. Moreover, TNF-α is a critical contributor to granuloma formation and is also involved in Mtb-induced apoptosis [7], [53], [54]. However, we found here that wild-type and mutant strains did not differ in the induction of TNF-α production by macrophages, indicating that ChoD affects only a subset of the functional activities of macrophages.

On the basis of the above observations and our experimental data, we hypothesize that ChoD is indispensable for Mtb effects on the TLR2-mediatated signaling pathway in the pathogenesis process. Mtb defective for the synthesis of ChoD stimulated macrophages to produce NO and ROS and limited the production of IL-10, resulting in reduced survival of bacteria inside macrophages. Our findings demonstrate that ChoD of Mtb participates in the virulence of tubercle bacilli and promotes pathogen survival in human macrophages.
